# Production and immunogenicity of a plant-produced beak and feather disease virus vaccine in Japanese quails

**DOI:** 10.1007/s00705-025-06352-z

**Published:** 2025-06-25

**Authors:** Goodman Mulondo, Mélie L. R. Buyse, Kimberley Labuschagne, David Jarvis, Albertha van Zyl, Edward P. Rybicki, Inga I. Hitzeroth, Sandiswa Mbewana

**Affiliations:** 1 Department of Molecular and Cell Biology, University of Cape Town, Cape Town, Rondebosch, 7700 South Africa; 2 Institute of Infectious Disease and Molecular Medicine, University of Cape Town, Cape Town, Rondebosch, 7700 South Africa; 3 Department of Chemical Engineering, University of Cape Town, Cape Town, Rondebosch, 7700 South Africa; 4 Liselo Labs, Howick, 3291 South Africa

**Keywords:** BFDV CP, *Nicotiana benthamiana*, quails, western blotting, antibodies (IgY)

## Abstract

Beak and feather disease virus (BFDV), a single-stranded DNA virus, infects endangered psittacine species, including the South African Cape parrot. The disease is highly contagious and can be transmitted through contact with contaminated faeces, crop secretions, and feather and skin dander. To date, there is no vaccine or cure available for BFDV. The production of an effective vaccine depends on having a production platform and methods that are both easy to use and capable of yielding a significant amount of protein that will induce a sufficient immune response. Therefore, the aim of this study was to produce a plant-based BFDV vaccine candidate and to evaluate its ability to elicit an immune response in birds.

Recombinant BFDV capsid protein (CP) was transiently expressed in *Nicotiana benthamiana* and purified using density gradient ultracentrifugation. Japanese quails were immunized with purified BFDV CP. Yolk-derived IgY was purified by water dilution and salt precipitation, and its specificity was verified by western blot analysis. The expression levels of the coat protein increased from non-detectable to an average accumulation of 1.58 mg/kg of fresh plant tissue biomass, and antibodies against BFDV CP were detected in both the blood and eggs of immunized quails, indicating that vaccination with BFDV CP successfully elicited a humoral immune response.

This study demonstrates that heterologous expression in plants is a viable method for producing BFDV CP. To the best of our knowledge, this is the first study to show the antibody response to a plant-produced BFDV antigen in a quail model. Given that the presence of anti-CP antibodies in infected birds is associated with immunity, this system can potentially be used to produce a vaccine against BFDV.

## Introduction

Beak and feather disease virus (BFDV) is a single-strand DNA virus belonging to the genus *Circovirus* of the family *Circoviridae*. It is the etiological agent of psittacine beak and feather disease (PBFD), which poses a significant threat to wild and captive psittacine bird species worldwide [14, 22, 32]. According to the latest update of the International Committee on Taxonomy of Viruses (ICTV), the family *Circoviridae* includes two genera, *Cyclovirus* and *Circovirus*. Circoviruses and the recently recognized cycloviruses are the smallest and simplest known pathogens causing disease in vertebrates [31]. As with all circoviruses, BFDV is predominantly species-specific [28, 38]. The single-stranded (ss) circular DNA genome of BFDV is approximately 2 kb in size [27] and has two major open reading frames (ORFs) that encode a single capsid protein (CP) and a replication-associated (rep) protein, which is responsible for the replication of the genome [5, 20]. The CP forms the viral capsid and is the main exposed antigen of the BFDV virion, which makes it the major target for development of a subunit vaccine [7, 33].

Due to the international trade in pet birds, PBFD remains one of the most relevant viral diseases in psittacine birds in both wild and captive psittacine populations in Asia, Africa, North America, Europe, and Australasia [13]. All psittaciforms are thought to be susceptible to the virus, with PBFD described in 78 different species thus far [13]. In South Africa, BFDV infections have been observed in captive and free-ranging endemic Cape parrots. As a result, about 10–20% of the psittacine captive breeding stock is lost annually due to BFDV [15, 37], posing a significant threat to this endangered species. Presently, no commercial vaccine is available to prevent PBFD [17, 21].

Recombinant protein expression systems used to express the BFDV CP include bacteria, yeast, mammalian cells, insect cell lines, and plants [23, 26]. Plants offer distinct advantages for vaccine production over other expression systems, as they are free from human and animal pathogens and are capable of post-translational modification of expressed proteins, which is often necessary for correct protein folding. The efficiency of expression of proteins in plants as recombinant vaccine candidates has improved greatly over the past 20 years, mainly because of a switch to transient expression. However, obtaining sufficient yields that are acceptable for commercial production (typically > 50 mg/kg) is far from easy and is still an ongoing process [6, 24, 29].

The BFDV CP has been expressed previously in *Nicotiana benthamiana* by Duvenage et al. [7] and by Regnard [25]; however, despite efforts to increase the protein yield, expression levels were not adequate for commercial production. Despite low expression levels, Regnard et al. [26] demonstrated that plant-expressed BFDV CP can self-assemble into virus-like particles (VLPs), which was considered a very promising step towards development of a recombinant vaccine. VLPs mimic the native virus structure but are non-infectious, as they do not contain any viral genetic material, making them much safer for use than manipulating the wild-type virus, and more efficient at eliciting a strong humoral and cellular immune response [6, 11, 34].

The aim of this study was to optimize the expression and purification of BFDV CP in *N. benthamiana* plants for the development of a plant-based vaccine candidate against BFDV. Quails were chosen for vaccination due to their ability to produce high yields of IgY antibodies in egg yolks, providing a non-invasive and cost-effective source of IgY antibodies. They were vaccinated with the purified BFDV CP, the immunogenicity of which was assessed by evaluating the specificity of serum and yolk-derived antibodies using western blot analysis.

## Materials and methods

### BFDV isolates and plant expression vectors

The sequence encoding the wild-type BFDV capsid protein (BFDV CP) of isolate BKS1ZA_84 (GenBank accession number GQ165756) was used in this study. This isolate was discovered previously in a budgerigar (*Melopsittacus undulates*) by Varsani et al. [37] and had been expressed successfully in plants by Regnard [25]. The South African budgerigar isolate BKS1ZA 84 was chosen in the previous study because it appeared to be closely related to the BFDV isolates from captive Cape parrots [25]. The CP was expressed using the vector pRIC3.0, an autonomously replicating plant expression vector based on the ssDNA virus bean yellow dwarf geminivirus (BeYDV) [24] that was specifically designed for cytoplasmic expression, as demonstrated in previous studies [25].

### Large-scale protein expression

For large-scale expression, each recombinant *Agrobacterium* construct was agroinfiltrated into the leaves of 5-week-old *N. benthamiana* plants. Previous studies have demonstrated that coexpression of the NSs silencing suppressor protein enhances recombinant expression of the gene of interest by suppressing posttranslational gene silencing in plants [35]. Therefore, the recombinant pRIC3.0 constructs were co-infiltrated with the silencing suppressor construct pBIN-NSs, which was provided by Marcel Prins from the Laboratory of Virology, Wageningen, The Netherlands. To assess background levels, the pRIC3.0 empty vector was used as a negative control.

The recombinant *A. tumefaciens* strain GV3101::pMP90RK, containing the BFDV CP in pRIC3.0, and *Agrobacterium* strain LBA4404, containing the pBIN-NSs construct, were grown overnight at 27°C. The cultures were grown in 10 mL of Luria-Bertani broth (LBB) medium supplemented with appropriate antibiotics. The medium for GV3101::pMP90RK was supplemented with rifampicin (50 µg/mL), kanamycin (30 µg/mL), and carbenicillin (50 µg/mL), while the medium for LBA4404 was supplemented with rifampicin (50 µg/mL) and kanamycin (30 µg/mL) [19]. This process was repeated over the next 2 days while transferring the cultures to larger flasks each day, with the last incubation step lacking rifampicin in the LBB. MgSO_4_ was added to the LBA4404 cultures to a final concentration of 2 mM to prevent cell clumping during incubation [19]. The absorbance (OD_600_) of 500-mL overnight cultures was measured using an Ultrospec10 Cell Density Meter (Amersham Biosciences, United Kingdom), and the cells were diluted with infiltration medium (10 mM MES, 10 mM MgCl_2_, and 200 µM acetosyringone, pH 5.6) to the required OD_600_ of 0.5 for the BFDV construct and 0.25 for pBIN-NSs. The appropriately diluted cultures were incubated at room temperature for 4 hours (h) to allow the acetosyringone to induce expression of the *vir* genes necessary for T-DNA transfer to the plant cells. Subsequently, both constructs were vacuum-co-infiltrated into 20–25 5-week-old whole plants. Plants were grown at 22°C with a 16 h/8 h light/dark cycle.

### Large-scale of CP extraction and purification

Large-scale protein extraction for each construct was first performed as described by Gunter et al. [12], and the purification procedure was further optimized using iodixanol (OptiPrep™, Sigma-Aldrich) density gradient centrifugation. Briefly, 100 g of plant leaves were harvested at 4 days postinfection (dpi) for WT CP and pRIC3.0. Leaves were homogenized in 120 mL of DB150 buffer (150 mM NaCl, 1 mM CaCl_2_, 0.001% Triton X-100, 0.25 M L-arginine, 10% glycerol (v/v), 10 mM Tris-HCl, pH 6.50), and 1x Complete EDTA-free Protease Inhibitor (Roche, Basel, Switzerland) using IKA T-25 ULTRA-TURRAX (Sigma-Aldrich, St. Louis, MO, USA). The crude extracts were clarified by centrifugation at 26,000 × *g* for 30 min at 4°C using a JA-14 rotor (Beckman Coulter Inc., Brea, CA) and an Avanti J25TI centrifuge (Beckman).

### Sucrose cushion density gradient centrifugation

After clarification, the supernatants were filtered through four layers of Miracloth (Merck, Kenilworth, NJ, USA) and loaded onto a sucrose cushion. A two-step sucrose gradient was made in Thinwall 38 mL Ultra-Clear ultracentrifuge tubes (Beckman) by underlaying 6 mL of 25% and 2 mL of 70% sucrose solution made in DB150 buffer. Each tube was loaded with 30 mL of clarified plant extract and centrifuged at 4°C for 4 h in a Beckman SW32Ti rotor at 175,000 × *g*. After centrifugation, 1-mL fractions were collected from the 25% and 70% sucrose layers and combined in a 50-mL centrifuge tube (WhiteSci, Cape Town, South Africa). The pooled fractions were dialyzed overnight against phosphate-buffered saline (1X PBS; 137 mM NaCl, 10 mM Na_2_HPO_4_, 2.7 mM KCl, and 2 mM KH_2_PO_4_, pH 7.4) at 4°C. Following dialysis, the samples were centrifuged at 26,000 × *g* for 20 min at 4°C using a JA-14 rotor (Beckman Coulter Inc., Brea, CA) and an Avanti J25TI centrifuge (Beckman).

### OptiPrep-based density gradient purification

Dialyzed samples were further purified using OptiPrep density gradients. OptiPrep is a sterile endotoxin-free solution of 60% iodixanol in water with a density of 1.32 g/mL [9, 10]. Briefly, OptiPrep Density Gradient Medium (Sigma-Aldrich, St. Louis, MO) was prepared at concentrations of 20%, 30%, 40%, and 50% in 1X PBS buffer. These gradients were then carefully underlaid with the concentrated, dialyzed plant extract in a Thinwall 38-mL Ultra-Clear ultracentrifuge tube. The tubes were centrifuged at 175,000 × *g* for 4 hours at 4°C using an Optima L-XP ultracentrifuge. Following centrifugation, 2-mL fractions were collected and analysed by 12% SDS-PAGE and western blotting.

### Western blot analysis

For the analysis of large-scale OptiPrep-purified protein, 5x sample application buffer (SAB; 2% SDS, 4.3% β-mercaptoethanol, 100 mM Tris-HCl [pH 7.5], 52% glycerol, and bromophenol blue [Bio-Rad]) was added to each sample to a final concentration of 1x before denaturing at 95°C for 10 min [30]. Proteins were resolved on 12% SDS polyacrylamide gels at 120 V, with equal volumes of sample loaded in each lane. A PageRuler Prestained Ladder (#SM0671, Thermo Fisher Scientific) was used as a molecular weight marker on all gels. After gel electrophoresis, proteins were transferred onto nitrocellulose membranes at 15 V for 1.5 h, using a Transblot semi-dry transfer cell (Bio-Rad). After transfer, the membranes were immersed in blocking buffer (5% non-fat dairy milk [NFDM], 1x PBS [pH 7.4], and 0.1% Tween-20) (PBST), for 30 min and then incubated overnight at 4°C, with shaking, in 1:2,000 rabbit anti-BFDV antibody diluted in blocking buffer (PBST). The following day, each membrane was washed four times for 15 min with blocking buffer and then incubated with a 1:2,000 dilution of anti-rabbit secondary antibody conjugated to alkaline phosphatase (Sigma-Aldrich) in blocking buffer for 1 h at 37°C with shaking at 100 rpm. The membranes were subsequently washed four times for 15 min with 1x PBST. Detection was performed with 5-bromo-4-chloro-3-indolyl-phosphate (BCIP) and nitroblue tetrazolium (NBT) phosphatase substrate (BCIP/NBT 1-component, KPL). In addition, to differentiate between RuBisCO, which is the most abundant plant protein and has a molecular weight of ~ 55 kDa, and the purified BFDV CP dimers (~ 56 kDa), a 1:5,000 dilution of commercial rabbit anti-RuBisCO antibody was used as the primary antibody, followed by a 1:10,000 dilution of alkaline-phosphatase-conjugated anti-rabbit secondary antibody. Polyclonal antibodies raised in rabbits immunized with BFDV CP produced in *E. coli* [3] were used to detect BFDV CP.

### Gel densitometry

CP expression was quantified by gel densitometry of Coomassie-blue-stained SDS-PAGE gels. A twofold serial dilution of bovine serum albumin (BSA, Sigma-Aldrich) was used as a reference to create a standard curve. An equal volume of each sample and standard was loaded onto a 12% SDS-PAGE gel for electrophoresis. For quantification of the WT CP, gels were stained for 1 h at 37°C with Coomassie brilliant blue R-250 (Sigma-Aldrich), followed by destaining at 37°C until the background was clear, leaving only the protein bands visible. The amount of protein relative to the BSA standards was measured using a SynGene reader, using GeneTool version 3.07.03 software (Synoptics Inc., UK).

### Animal study

#### Immunization of quails

Immunization of quails was performed according to UCT Faculty of Science Animal Ethics Committee (SFAEC) approval no: 2021/V10/IH/A, the Department of Livestock Services, Maseru, Lesotho (protocol number: LISELOLABS_RFA_#0129), and Department of Agriculture Land Reform and Rural Development (DALRRD) of South Africa Section 20 approval 12/11/1/7/3 (2082 LH). The Japanese quails were maintained and bred at the animal farm facilities of Liselo Labs in Maseru, Lesotho. Two groups of five female Japanese quails (*Coturnix japonica)*, aged five to six weeks and weighing 160–250 g each, were used in this study. A sample with a total volume of 200 µl, containing 10 µg of final density-gradient-purified BFDV CP antigen in 1X PBS, pH 7.4, was mixed with 200 µl of Freund’s complete adjuvant and then injected intramuscularly in the pectoral muscle at one site only. In comparison, a negative control group was injected with purified plant material containing empty vector only, purified in parallel with BFDV CP. The first vaccination was done on day 0, and two booster vaccinations were administered on days 14 and 28. A third and final booster was administered 3 months after the second booster, using 20 µg of purified BFDV CP. For serum antibody analysis, blood was collected 42 days after the first vaccination and one week after the third booster.

#### Purification of IgY and serum

IgY was purified from eggs collected the first week after the second and third booster. Eggs laid by quails from the same immunized group were grouped and stored at 4°C before processing. IgY was purified according to a protocol adapted from Hodek et al. [16]. Briefly, eggshells were carefully opened using specifically designed quail-egg-cutting scissors, and the egg yolks were separated from the egg whites, which were discarded. The yolks were diluted in seven volumes of tap water, acidified to pH 5.0 ± 0.1 with 1 M HCl, and frozen at -20°C overnight. The frozen yolk mixtures were transferred to clean funnels with one layer of folded Macherey-Nagel MN 615 ff Grade Filter Paper, diameter 240 mm (Thermo Fisher Scientific) and allowed to filter as they thawed. The antibodies in the filtrates were precipitated out of solution by adding 8.8% (w/v) NaCl and adjusting the pH to 4.0 ± 0.1 with 1 M HCl. The mixtures were allowed to precipitate by static incubation for 2 h at room temperature and centrifugation at 3,700 × *g* for 20 minutes. The supernatants were carefully removed and discarded, and the pellets were resuspended in 1X PBS and stored at -20°C until used for western blot analysis to determine the specificity of the IgY antibodies to BDFV CP.

#### Determination of IgY specificity

Four different western blots were used to determine the specificity of quail-produced IgY antibodies. Iodixanol-purified BFDV CP antigen and empty vector pRIC3.0, used as a negative control, were resolved in parallel on 12% SDS polyacrylamide gels at 120 V, with equal volumes of sample loaded in each lane. In parallel, negative control blots were run for comparison, using IgY antibodies from birds inoculated with a purified empty pRIC3.0 sample. The membranes were incubated separately overnight at 4°C with shaking at 100 rpm in either 0.5 mg of yolk-derived IgY antibody per ml or a 1:1000- or 1:2000-diluted serum IgY antibody diluted in blocking buffer. As a positive control, the membrane was probed with a 1:2000 dilution of rabbit anti-BFDV CP antibody. The secondary antibodies used were a 1:5000 dilution of anti-chicken IgY (IgG) (whole molecule) alkaline phosphatase conjugate (Sigma-Aldrich) for the IgY blots and a 1:10,000 dilution of anti-rabbit alkaline phosphatase conjugate (Sigma-Aldrich) for the positive control blot.

## Results

### Expression and purification of BFDV CP

**Fig. 1:**
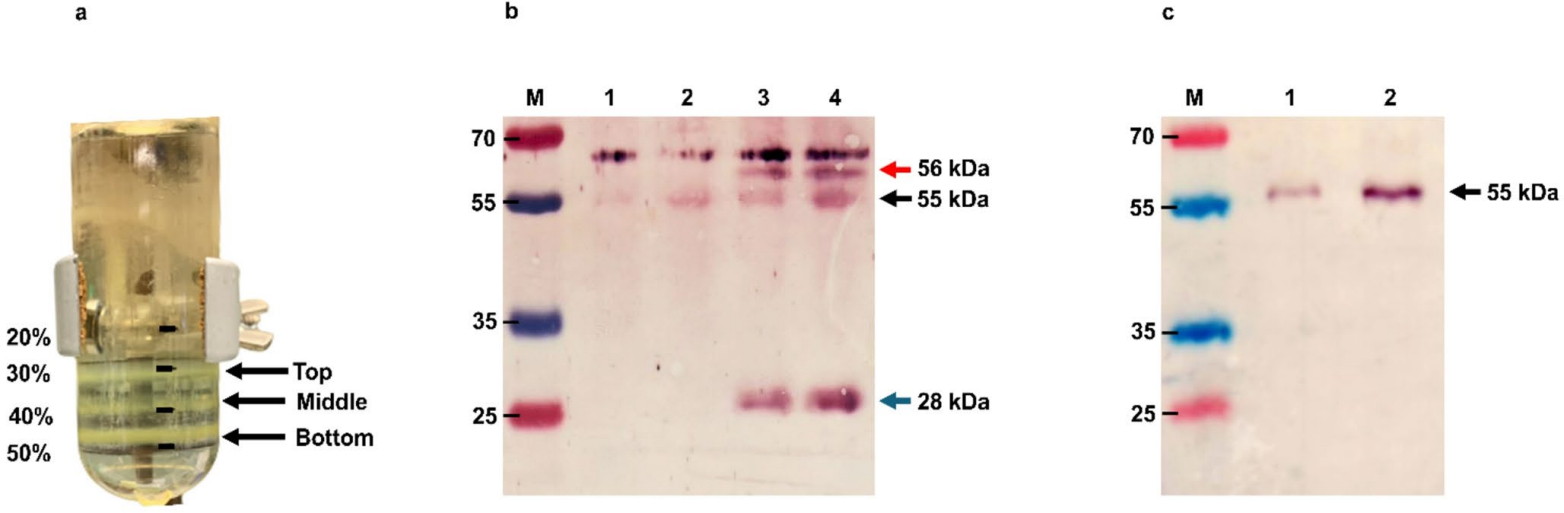
Iodixanol density gradient ultracentrifugation purification of BFDV CP. (**a**) Image of a 14 × 89 mm ultracentrifuge tube after 4 hours of ultracentrifugation. Three bands were observed between the 30% and 50% iodixanol gradient layers. (**b**) Western blot analysis of plant-expressed BFDV CP, purified using a sucrose cushion followed by OptiPrep density gradient ultracentrifugation. 1:2000-diluted rabbit anti-BFDV antiserum was used as the primary antibody, and a 1:10,000-diluted anti-rabbit-IgG alkaline phosphatase-conjugate was used as the secondary antibody. Lane M, molecular weight marker; lane 1, diluted pRIC 3.0 fraction extracted from the middle band; lane 2, undiluted pRIC 3.0 fraction extracted from the middle band; lane 3, diluted BFDV fraction extracted from the middle band; lane 4, undiluted BFDV fraction extracted from the middle band. Blue arrow, BFDV CP (28 kDa); red arrow, BFDV CP dimer (56 kDa); black arrow, plant RuBisco (55 kDa). (**c**) Western blot analysis to distinguish the bands corresponding to RuBisco and BFDV CP dimers. A 1:5000-diluted commercial rabbit anti-RuBisco antibody was used as the primary antibody, and a 1:10,000-diluted alkaline-phosphatase-conjugated anti-rabbit antibody was used as the secondary antibody. Lane M, molecular weight marker (PageRuler Prestained Protein Ladder, Thermo Fisher Scientific); lanes 1 and 2, pRIC 3.0 and BFDV CP, respectively, after final iodixanol purification. Black arrow, plant RuBisco (55 kDa)

The extraction buffer used previously by Gunter et al. [12] resulted in higher protein yield than that attained using other methods. Recombinant BFDV CP was purified using a two-step protocol. First, the protein was subjected to sucrose cushion centrifugation to remove cellular debris and to concentrate the target protein. Second, OptiPrep (iodixanol) density gradient ultracentrifugation, which is especially well-suited for concentrating and separating expressed proteins from residual plant impurities, was used to further concentrate the purified proteins. Following ultracentrifugation, purified BFDV CP was extracted from the interface between the 30% and 40% iodixanol layers (Fig. 1a). Western blot analysis further confirmed the presence of BFDV CP, with a molecular weight of 28 kDa, along with its dimer at 56 kDa (Fig. 1b, lanes 3 and 4). In contrast, no bands corresponding to BFDV CP proteins were observed in the pRIC3.0 negative control (Fig. 1b, lanes 1 and 2).

**Fig. 2:**
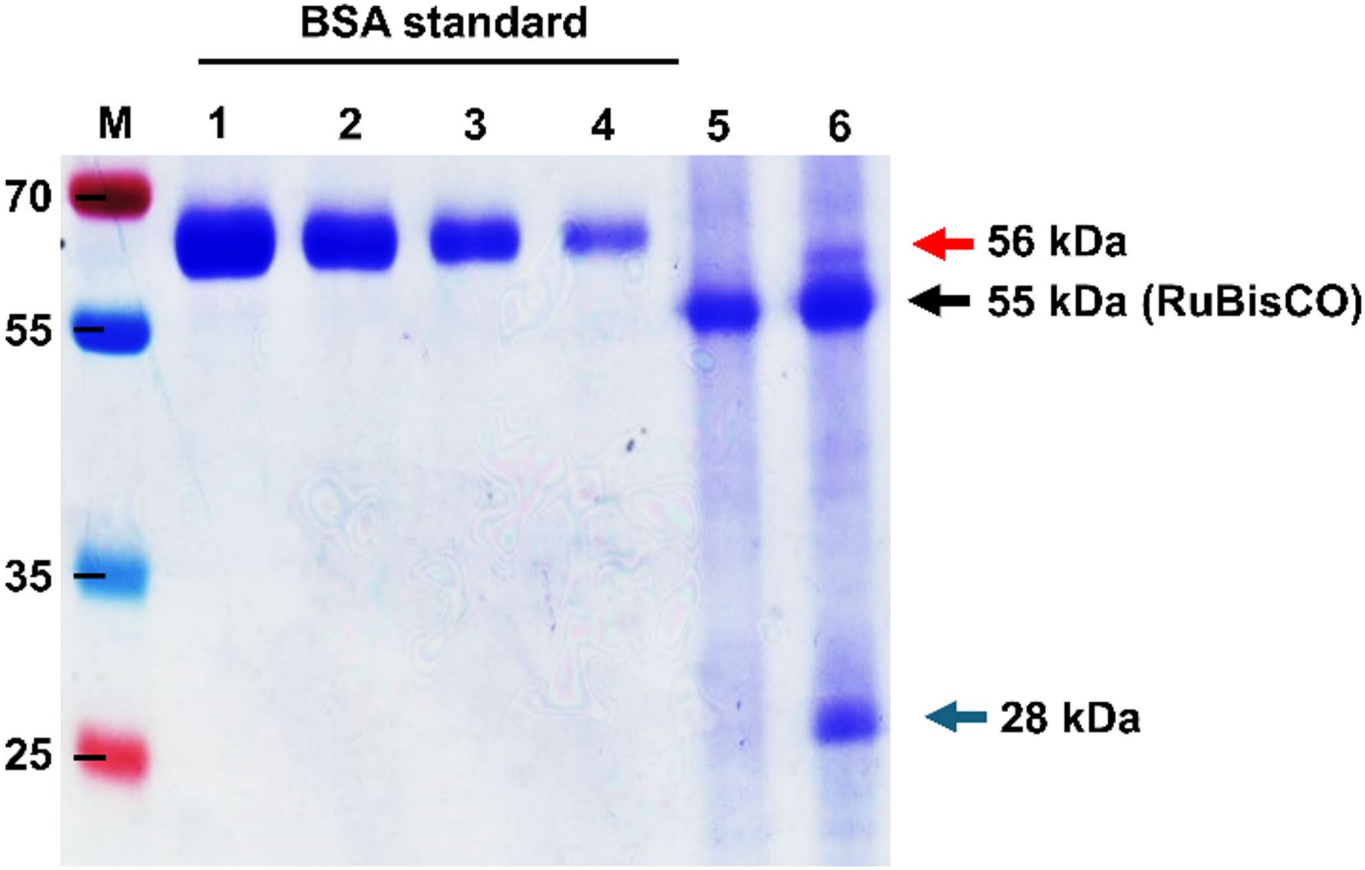
Protein quantification images of iodixanol-gradient-purified plant-expressed BFDV CP. Lane M, molecular weight marker; lanes 1–4, BSA protein standards at concentrations of 3.125 µg, 1.562 µg, 0.781 µg, and 0.390 µg, respectively; lane 5, vector-only negative control; lane 6, undiluted VLP sample. Blue arrow, BFDV CP (28 kDa); red arrow, BFDV CP dimer (56 kDa); black arrow, plant RuBisCO (55 kDa)

The purified samples were found to be free of bacterial contamination before animal experiments (data not shown). In addition, to determine if the detected bands consisted of co-purified RuBisCO plant protein (~ 55 kDa) or purified BFDV CP dimers (~ 56 kDa), an undiluted fraction of the negative control pRIC3.0 (lane 2) and BFDV CP (lane 4) from Fig. 1b were analysed on a western blot probed with anti-RuBisCO antibody (Fig. 1c). The results indicated that the bottom band observed at 55 kDa (Fig. 1c) corresponded to RuBisCO. This confirmed that the higher-molecular-weight band observed above RuBisCO, with a size of approximately 56 kDa, represents the BFDV CP dimer, consistent with the findings of Regnard [25]. The yield in this study was 1.58 mg of purified BFDV WT CP per kg of fresh-weight biomass (Fig. 2).

### Production of BFDV-specific antibodies in quail blood

**Fig. 3:**
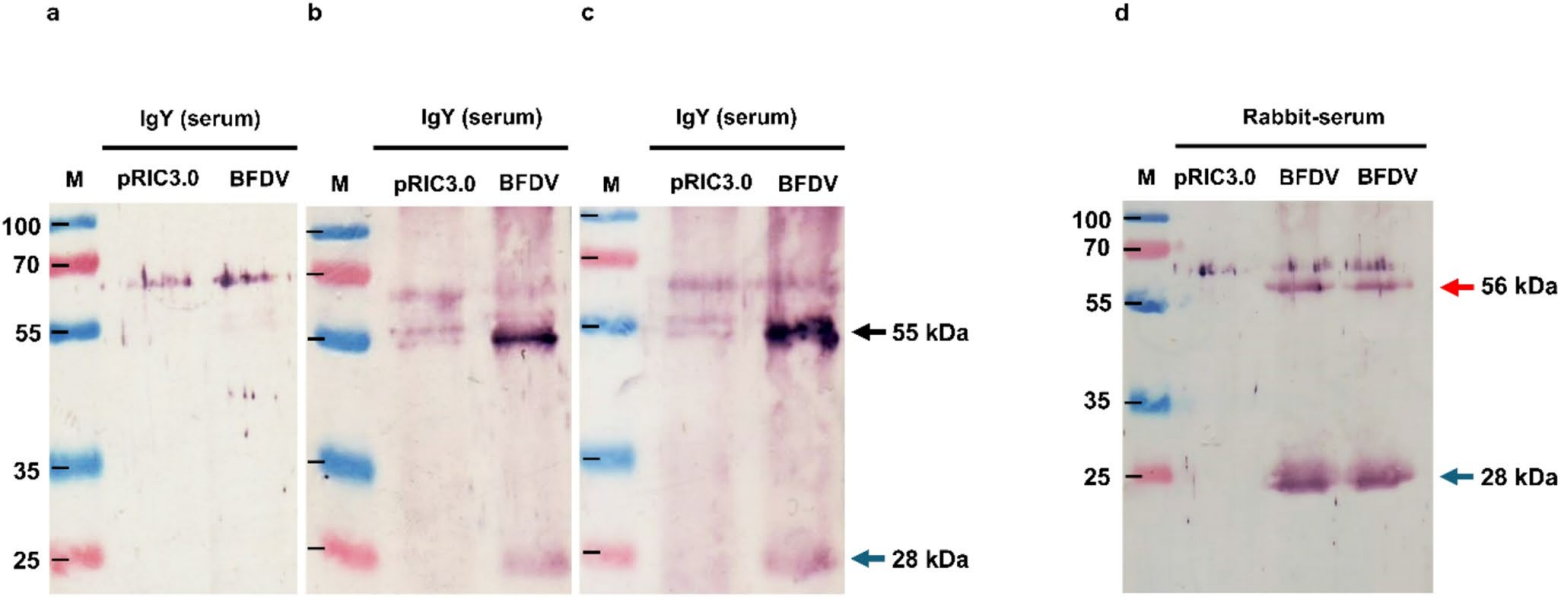
Western blot analysis of serum BFDV-specific IgY antibodies. Lane M, molecular weight marker; pRIC3. 0, plant-expressed empty vector pRIC3.0; BFDV CP, plant-expressed BFDV CP protein. For all blots, 30 µl of either pRIC 3.0 or BFDV (1 µg) was loaded. (**a**) For quails inoculated with empty pRIC3.0, the primary antibody used was 1:1000-diluted quail serum IgY. (**b**) For quails inoculated with BFDV CP, the primary antibody used was 1:1000-diluted quail serum IgY (collected after the third boost). (**c**) For quails inoculated with BFDV CP, the primary antibody used was 1:2000-diluted quail serum IgY (collected after the third boost). (**d**) For the positive control blot, the primary antibody used was 1:2000 rabbit anti-BFDV CP serum. For blots a, b, and c, a 1:5000 dilution of rabbit anti-chicken IgY (IgG) (whole molecule) alkaline-phosphatase-conjugated antibody was used as the secondary antibody. For blot d, a 1:10,000 dilution of alkaline-phosphatase-conjugated anti-rabbit IgG was used as the secondary antibody

To determine whether BFDV CP antigen was able to induce the production of specific serum IgY antibodies in immunized quails, blood was collected 42 days after the primary vaccination, and seven days after the final booster, and serum obtained from these samples was used as the primary antibody for the detection of plant-expressed BFDV CP on western blots. On each blot, samples purified from plants infiltrated with empty vector pRIC3.0 were used as a negative control. The blots were probed with a 1:1000 or 1:2000 dilution of quail serum. However, quail antiserum collected after the second booster did not detect the plant-purified BFDV CP at a 1:1000 or 1:2000 dilution (not shown). As a negative control, blots were probed with antisera from birds inoculated with a purified extract from a plant infiltrated with the empty pRIC3.0 vector (Fig. 3a). However, antisera collected after the third booster were able to detect both the plant-produced BFDV CP and the BFDV CP dimer (Fig. 3b and c). A western blot probed with 1:2000-diluted rabbit anti-BFDV CP serum was used as a positive control (Fig. 3d).

### Production of BFDV-specific IgY in egg yolk

**Fig. 4:**
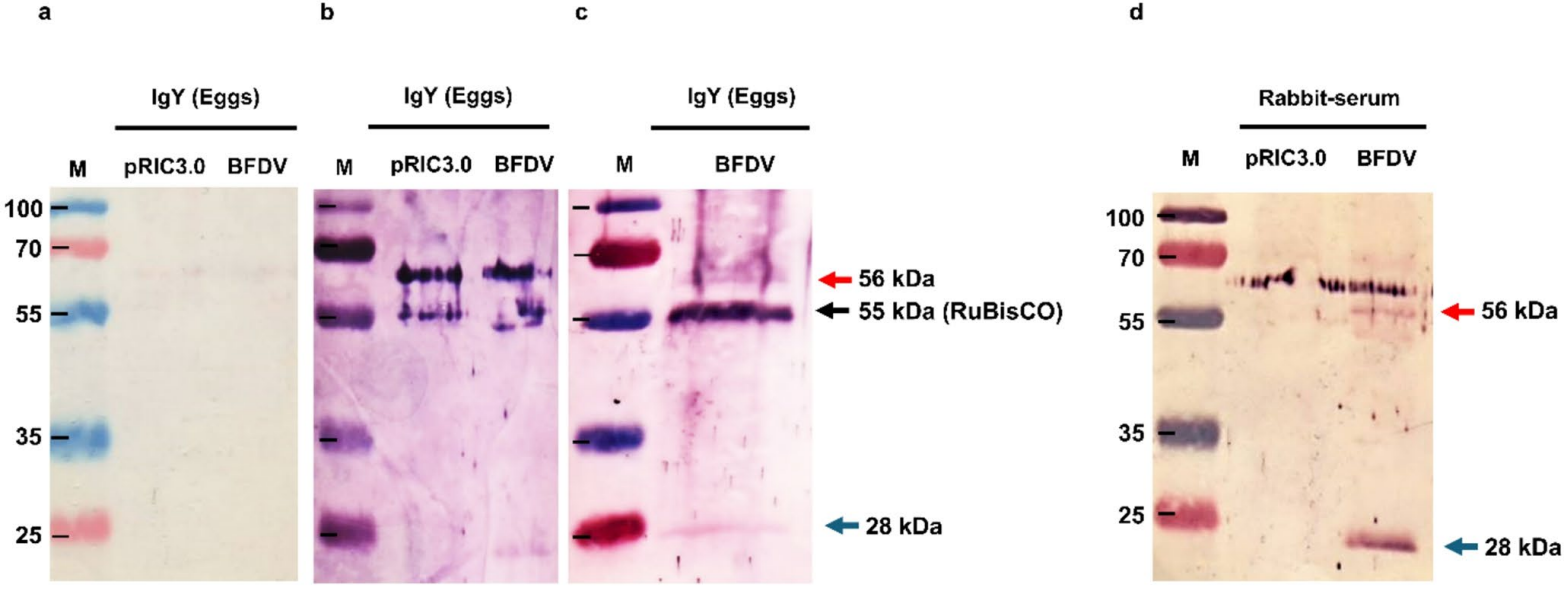
Western blot analysis of BFDV-specific yolk IgY antibodies. Lane M, molecular weight marker; pRIC 3.0, plant-expressed empty vector pRIC3.0; BFDV CP, plant-expressed BFDV CP. For blots a, b, and d, 30 µl (1 µg), and for blot c, 60 µl (2 µg) of either pRIC3.0 or BFDV was loaded. (**a**) For quails inoculated with empty pRIC3.0, the primary antibody used was 0.5 mg/mL yolk-derived IgY. (**b** and **c**) For quails inoculated with BFDV CP, the primary antibody used was 0.5 mg/mL yolk-derived IgY (collected after the second booster). (**d**) Positive control blot. The primary antibody used was 1:2000-diluted rabbit anti-BFDV CP serum. For blots a, b, and c, a 1:5000 dilution of rabbit anti-chicken IgY (IgG) (whole molecule) alkaline-phosphatase-conjugated antibody was used as the secondary antibody. For blot d, a 1:10000 dilution of alkaline-phosphatase-conjugated anti-rabbit IgG was used as the secondary antibody

To determine whether the purified yolk IgY was specific for BFDV CP, western blots were loaded with either 30 µl (1 µg) (Fig. 4b) or 60 µl (2 µg) (Fig. 4c) of expressed purified plant BFDV CP, and the pRIC3.0 empty vector was probed with 0.5 mg of the purified yolk IgY sample per mL. Yolk IgY purified from eggs collected after the second booster was able to detect BFDV CP (Fig. 4b and c). No similar bands were detected on the negative control blot probed with yolk IgY from quails inoculated with the empty pRIC3.0 sample (Fig. 4a). However, yolk IgY purified one week after the third and final booster was unable to detect BFDV CP (not shown). To confirm that the bands detected with yolk IgY were indeed BFDV CP, a 1:2000 dilution of a rabbit anti-BFDV CP serum was used as a positive control (Fig. 4d). Bands at the expected positions for the BFDV CP and BFDV CP dimer were observed, confirming the specificity of the yolk-derived IgY antibodies.

## Discussion

Disease prevention through effective vaccination protocols plays an important role in veterinary disease management. Currently, no commercial BFDV vaccine is available, and the development of an effective vaccine against BFDV could help to prevent economic losses encountered by aviculturists and aid parrot conservation efforts in South Africa and the rest of the world [8, 25, 33]. Systems for expression of recombinant CP have become the focus of BFDV vaccine development. CP has been successfully produced in several expression systems, but commercial large-scale vaccine production has not yet been achieved due to low yields of CP, resulting in production costs that are prohibitively high for scaling up [23, 33]. Therefore, the aims of this study were to optimize the production of BFDV CP to ensure its suitability for vaccine development and to identify a suitable animal source of CP-specific antibodies for therapeutic purposes.

For extraction of CP from plant tissue, we used DB150 buffer, which was used previously by Gunter et al. for purification of porcine circovirus (PCV)-2 VLPs [12]. This resulted in the extraction of BFDV CP while minimising the co-extraction of plant proteins (Fig. 1b). The presence of L-arginine together with Triton X-100 in the DB150 extraction buffer has been shown to enhance protein solubilization [2], while the presence of glycerol improves protein stability and prevents aggregation [36]. These combined effects most likely contributed to the efficiency of this method, corresponding to the results of a previous study investigating the optimization of BFDV CP expression in *Escherichia coli*. In that study, a 15- to 20-fold higher level of protein expression was observed when using an extraction buffer containing Triton X-100 and glycerol [21]. The results of the present study suggest a possible improvement on the methods used by Regnard et al. [26] in which BFDV CP purified from *N. benthamiana* using a sucrose cushion and CsCl density gradient centrifugation could not be detected on an SDS-PAGE gel stained with Coomassie blue dye, suggesting that the amount of protein was below the detection threshold of 25 ng per band. Those results indicated a yield of less than 5 mg per kg of fresh leaves and might also have been lower than the 1.58 mg/kg obtained in the current study, in which BFDV CP was detectable on an SDS-PAGE gel (Fig. 2). While the concentration of BFDV CP in the current study was significantly higher than that reported by Regnard et al. [26], the overall yield was lower than the 6.5 mg/kg reported in the PCV-2 study [12]. Consequently, although the two-step purification method reported in the present study was deemed very successful, the overall protein yield of 1.58 mg of CP per kg of fresh leaves was considered poor. However, low yields of CP do not necessarily pose a problem for evaluating immunogenicity, as smaller antigen doses could potentially be highly effective in triggering a protective immune response.

Japanese quails were inoculated with purified BFDV CP to stimulate IgY antibody production. Although we were not able to detect plant-produced BFDV CP using BFDV-specific IgY serum antibodies collected 14 days after the second booster vaccination (data not shown), antisera collected seven days after the third and final booster were able to detect BFDV CP and BFDV CP dimers (Fig. 3b and c). On the other hand, purified yolk IgY, at a concentration of 0.5 mg/mL, was able to detect both plant-purified BFDV CP and CP dimers two weeks after the second booster (Fig. 4b and c), but no bands were detected one week after the final vaccination (data not shown). It has been documented previously that the IgY concentration in egg yolk is generally higher than that found in serum [1] due to active transport from the hen to the yolk, which is important for conferring passive immunity to newly hatched chicks. In chickens, it has been reported that the level of yolk IgY antibodies can rise within one week to an amount equivalent to that found in 90 to 100 mL of serum or 180 to 200 mL of whole blood [18]. This could explain why we were unable to detect BFDV CP with serum IgY collected two weeks after the second booster inoculation (data not shown) but were able to detect a significant amount of BFDV CP on western blots probed with yolk IgY collected at the same time (Fig. 4b and c). Unexpectedly, while the third booster, which was administered three months after the second booster, generated enough serum IgY for detection of BFDV CP after one week, yolk IgY levels were insufficient for detection, in contrast to IgY collected two weeks after the second booster. It has been shown that the transovarial passage of IgY from the hen to the egg yolk takes between 3 and 6 days [4]. Therefore, it is likely that not enough antibodies were present in the yolk at the time of collection of the final eggs; however, the final vaccination did stimulate the production of a notable amount of serum IgY compared to the second booster. This is not surprising, as an adequate immune response relies on five main factors: the dose and molecular weight of the antigen, the adjuvant, how it is administered, how many boosters are administered, and the interval between doses [4], and these factors most certainly also influenced the results presented here. It is also possible that the presence of RuBisCO in the plant-produced proteins used to formulate the vaccine may have skewed the immune response towards RuBisCO.

Several different purification methods were explored to eliminate or reduce RuBisCO contamination, but these were not successful (data not shown). Ammonium sulfate precipitation resulted in coprecipitation of RuBisCO with BFDV CP at all tested concentrations, pH-based precipitation using the isoelectric point (pI) of RuBisCO caused co-precipitation of BFDV CP at all tested time points, ion-exchange chromatography based on the pI of BFDV CP did not yield detectable BFDV CP in any of the collected fractions due to the low concentration of BFDV CP, and sucrose cushion centrifugation alone also failed to yield detectable BFDV CP. It was only after iodixanol density gradient purification following sucrose cushion centrifugation that BFDV CP was obtained at a sufficiently high concentration to be detectable.

## Conclusions

In this study, we optimized the transient expression of BFDV CP in *N. benthamiana* plants, increasing levels from non-detectable to an average of 1.58 mg/kg of fresh plant tissue biomass. To the best of our knowledge, this work is the first study to evaluate both the humoral response against BFDV CP in quails and the production of antibodies in yolk. Quail anti-BFDV CP IgY antibodies from both the yolk and serum were specific for BFDV CP when used as primary antibodies for immunoblotting. These results demonstrate that BFDV CP has the potential to be used as a vaccine for stimulating an antibody-mediated immune response against BFDV infection and also show that quails are a simple, inexpensive, and fast expression system for antibodies that can be used for both immunoassays and immunotherapeutic purposes. We conclude that production in plants could be a viable means of making BFDV vaccines. Future work should consider alternate methods for more-efficient depletion of RuBisCO from BFDV CP preparations prior to vaccine formulation, evaluating the neutralizing capacity of antibodies produced by the candidate vaccine, and determining if the antibody-mediated immune response is protective against BFDV infection.
